# A Thousand Aphorisms on the Birth of Man

**Published:** 1845-04

**Authors:** 


ART. IV.—*Tausend Aphorismen über die Geburt des Menschen.* Von JOSEPH HERMANN SCHMIDT, M.D. &c.—*Berlin*, 1844.

*A Thousand Aphorisms on the Birth of Man.* By DR. J. H. SCHMIDT. —*Berlin*, 1844. 8vo, pp. 246.

IT is very annoying to the patience of a reviewer to wade through a book which is written in a peculiarly difficult style and, withal, of no very humble pretensions, to be led on from page to page of his wearisome journey, in the hope of finding something worth notice; and at last, when he reaches the end of his trial, not less worn out in mind than in patience, he is obliged to close it with the feeling that the task has been an utterly fruitless one, and for the best reason, because there has been no fruit to gather.

Such is the case with the book now before us. It is a singular specimen of pompous transcendental pebble-picking, mystifying as well as misleading a perhaps learned, but certainly by no means practical mind into all sorts of absurd extravagances.

It cannot be denied that the author is an original thinker, for it would puzzle the wisest heads to find the analogies, parallelisms, similes, and antitheses in which his thoughts seem to revel on every subject and occasion.

We can scarcely call his paragraphs aphorisms, for we cannot say that we have found anything in the whole book which deserves the name of a good practical aphorism.

As a specimen of style we give the following:

"The muscular fibres develop themselves in a vascular system in quantitative succession of stages by the intermediate stage of the half fibres, in the uterine system, also in *periodical* succession of stages by *the intermediate stage of the half fibres*, but in the one case as in the other, the *functional pulse* develops itself parallel with the formation of the substratum, (of the fibres)." !! (§ 64.)

As a specimen of his analogies, &c. we will quote his 256th aphorism.

"Generally speaking, there exists a wonderful analogy between the formation of the brain and of the ovum, although not with strict parallelism of the individual portions. The chorion is the dura mater, the amnion the arachnoidea, the decidua the pia mater of the ovum. The pia mater forms the inner covering of the brain, in the ovum the outer one, which latter is owing to the ovum being through it connected with a still greater covering, (the mother.)" !!

At another time he compares the uterus to an intestinal canal, the broad ligaments of which are the mesentery; but his grand triumph in the way of parallelisms and analogues is where he considers menstruation, pregnancy, and lactation, to be a species of physical trinity, like magnetism, electricity, and chemical action. Wherefore he calls up other triune combinations, medicine, surgery, and midwifery; nervous, vascular and cellular tissue; and winds up, most appropriately with "church, state, and school," rather shocking his conservative readers by putting the state first. What a pity it is that our friend Alexis had not visited Paderborn, from whence our author dates his lucubrations, before he came to London!

Dr. Schmidt promises us another batch of a thousand aphorisms; but we have quite satisfied ourselves, and, we also hope, our readers that any further notice of the Paderborn oracles, will be unnecessary.

